# Cancer-related cognitive impairment as a key contributor to psychopathology in cancer survivors: implications for prevention, treatment and supportive care

**DOI:** 10.1007/s00520-024-08696-9

**Published:** 2024-07-02

**Authors:** Darren Haywood, Melissa Henry, Evan Dauer, Oscar Lederman, Morgan Farley, Ashley M. Henneghan, Moira O’Connor, Michael Jefford, Susan L. Rossell, Nicolas H. Hart

**Affiliations:** 1 Human Performance Research Centre, INSIGHT Research Institute, Faculty of Health, University of Technology Sydney (UTS), Moore Park, Sydney, NSW 2030 Australia; 2 Department of Mental Health, St. Vincent’s Hospital Melbourne, Fitzroy, VIC Australia; 3 Department of Psychiatry, Melbourne Medical School, Dentistry and Health Sciences, University of Melbourne, Parkville, VIC Australia; 4 School of Population Health, Faculty of Health Sciences, Curtin University, Bentley, WA Australia; 5 Department of Otolaryngology−Head and Neck Surgery, McGill University Health Centre, Montreal, QC Canada; 6 Department of Oncology, McGill University Health Centre, Montreal, QC Canada; 7 Lady Davis Research Institute, McGill University, Montreal, QC Canada; 8 Research Institute of McGill University Health Centre, Montreal, QC Canada; 9 School of Health Science, Faculty of Medicine and Health, University of New South Wales, Sydney, NSW Australia; 10 School of Nursing, University of Texas at Austin, Austin, TX USA; 11 Department of Oncology, Dell Medical School, The University of Texas at Austin, Austin, TX USA; 12 Department of Health Services Research, Peter MacCallum Cancer Centre, Melbourne, VIC Australia; 13 Australian Cancer Survivorship Centre, Peter MacCallum Cancer Centre, Melbourne, VIC Australia; 14 Sir Peter MacCallum Department of Oncology, University of Melbourne, Parkville, VIC Australia; 15 Centre for Mental Health and Brain Sciences, Swinburne University of Technology, Hawthorn, VIC Australia; 16 Caring Futures Institute, College of Nursing and Health Sciences, Flinders University, Adelaide, SA Australia; 17 Exercise Medicine Research Institute, School of Medical and Health Sciences, Edith Cowan University, Perth, WA Australia; 18 Cancer and Palliative Care Outcomes Centre, Faculty of Health, Queensland University of Technology (QUT), Brisbane, QLD Australia; 19 Institute for Health Research, University of Notre Dame Australia, Perth, WA Australia

**Keywords:** Cancer-related cognitive impairment, Mental health, Psychopathology, Supportive Care, Cancer

## Abstract

A significant proportion of cancer survivors will experience some form of mental health compromise across domains including mood, anxiety, psychosis, eating disorders, and substance use. This psychopathology within cancer survivors is related to a range of negative outcomes and can also have a substantial negative impact on quality of life. Along with psychopathology, cognitive impairments are also commonly experienced, resulting in deficits in memory, reasoning, decision-making, speed of processing, and concentration, collectively referred to as cancer-related cognitive impairment (CRCI). Within the non-oncology literature, cognitive deficits are consistently demonstrated to be a key transdiagnostic aetiological feature of psychopathology, functionally contributing to the development and perpetuation of symptoms. Whilst there is an acknowledgement of the role mental health concerns might play in the *development* of and perception of CRCI, there has been limited acknowledgement and research exploring the potential for CRCI to functionally *contribute* toward the development of transdiagnostic psychopathology in cancer survivors beyond simply psychosocial distress. Given the theoretical and empirical evidence suggesting cognitive deficits to be an aetiological factor in psychopathology, we provide a rationale for the potential for CRCI to be a factor in the development and perpetuation of transdiagnostic psychopathology in cancer survivors. This potential functional association has significant implications for risk identification, prevention, treatment, and supportive cancer care approaches regarding psychopathology in cancer survivorship. We conclude by providing directions for future research in this area.

## Mental health in *cancer* survivorship

Cancer survivors (i.e. individuals with a history of cancer, from diagnosis through to the end-of-life, including all types, stages, and trajectories) are at a significantly greater risk of experiencing mental ill-health when compared to the general population [1]. It is commonly reported that cancer survivors often experience heightened psychological distress, depression, anxiety [1], and symptoms relating to post-traumatic stress disorder (PTSD; [2]). However, cancer survivors frequently exhibit a wider spectrum of psychopathology, with for example significantly heightened rates of symptoms related to psychotic disorders, substance use disorders, personality disorders [1], eating disorders [3], and obsessive–compulsive disorder [4]. A recent systematic review and meta-analysis found a notably high, 1.46) hazard ratio for developing mental illness after cancer [5]. Further, many cancer survivors experience a co-occurrence of psychopathologies [1, 6]. For example, estimates indicate that almost half of cancer survivors who have received a diagnosis of a mental disorder, will also have met the criteria for a second, and almost half of those cancer survivors will have met a diagnosis for a third mental disorder [e.g. 6]. Psychopathology within cancer survivors is related to a range of negative outcomes and can also have a substantial negative impact on quality of life [1, 7, 8].

The co-occurrence of psychopathologies within cancer survivorship and the general population is well established [9, 10]. In general, the transdiagnostic hypothesis postulates that there may be underlying common mechanisms to psychological symptoms and taxonomies, contributing to the onset, maintenance, clinical management, and recovery from mental health concerns [11]. Decades of data-driven research support the more accurate viewpoint of understanding mental health as existing dimensionally along spectrums rather than as distinct categories [11, 12]. For example, the Hierarchical Taxonomy of Psychopathology (HiTOP) model conceptualizes psychopathology as dimensional and consisting of higher (e.g. Subfactors, Spectra, and Superspectra) and lower-level (Symptoms, and Homogeneous Symptom Components and Maladaptive Traits) domains that capture the more shared or unique characteristics of psychopathology [12].

Defining mechanisms that actively contribute to the emergence and persistence of the diverse array of psychopathology in cancer survivors has significant implications for improvements to psychopathology risk identification, mitigation, and treatment approaches. So far, hypothesized contributing factors in cancer survivorship have been primarily focused on factors including fear of cancer recurrence or progression, financial toxicity, and guilt [13, 14]. While establishing plausible etiological mechanisms that account for the broader spectrum of psychopathology encountered by cancer survivors has been challenging, our article argues for the consideration of cognitive deficits as a particular etiological feature of psychopathology.

### Cognitive deficits as an etiological feature of psychopathology

The NIMH Research Domain Criteria (RDoC) framework is an epistemic framework that breaks down mental health complexity into six overarching domains: positive valence systems, negative valence systems, cognitive systems, systems for social process, arousal/modulatory systems, and sensorimotor systems [15]. These domains can reflect themselves in genes, molecules, cells, circuits, physiology, behaviour, self-report, and/or paradigms. Considering the high levels of distress in patients with cancer, the potential for chronic stress persisting into survivorship, and the effects of treatments, one may postulate an enhancement and interinfluence of higher-level domains of psychopathology.

Cognitive functioning is thus one domain that may functionally contribute to the development and perpetuation of a wide range of psychopathology for cancer survivors. Within the non-oncology literature, there is robust support from psychological, cognitive, neurological, immunological, and genetic theories, along with empirical evidence, that consistently underpins the proposition that cognitive deficits play a substantial role in the development and perpetuation of transdiagnostic psychopathology see [11, 16]. This is thought to occur through two pathways: *Pathway 1*; individuals with cognitive deficits may face some challenges in confronting, interpreting, and reinterpreting negative or irrelevant thoughts, events, and perceptual stimuli in daily life [11, 17]. *Pathway 2*; individuals with cognitive deficits may be more susceptible to negative life events across educational, occupational, and social domains due to their cognitive challenges, indirectly contributing to the development and perpetuation of psychopathology [11, 17]. The emerging evidence suggests that multiple cognitive functions may interact and functionally contribute toward psychopathology [18, 19]; however, deficits in speed of information processing may be especially functionally related to mental health challenges [11, 20–23]. Across both adult and youth samples, speed of information processing has been found to account for unique variance in transdiagnostic psychopathology over and above that accounted for by general cognitive functioning [21, 24].

### Cancer-related cognitive impairment and psychopathology

Up to 75% of cancer survivors report experiencing cognitive impairment, known as cancer-related cognitive impairment (CRCI). These impairments affect diverse cognitive domains, including memory, reasoning, decision-making, speed of processing, and concentration [25, 26]. The onset of these impairments can occur during cancer development, or become noticeable during the treatment or the post-treatment phase, and can last up to 20 years post-treatment and following cancer elimination [26–28]. Various psychosocial and biological accounts exist regarding the development of CRCI, and a range of supportive and treatment options have been, and continue to be, developed. However, ultimately CRCI has been shown to have significant effects on an individual’s daily life, relationships, occupational functioning, and social functioning [27, 28].

**Fig. 1:**
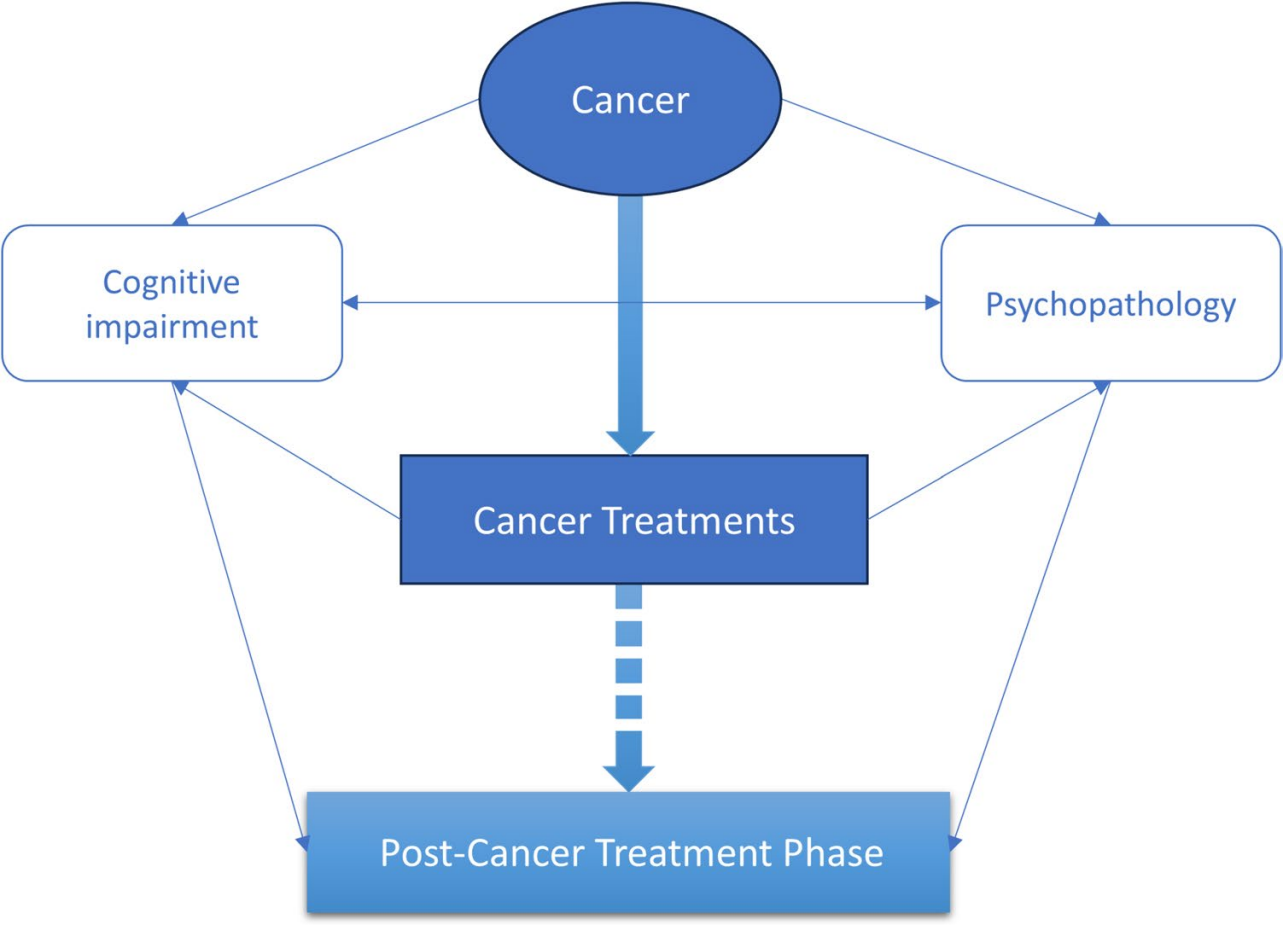
Proposed functional associations between cancer, cancer treatments, cognitive impairment, and psychopathology over the cancer continuum. ***Note.*** The broken arrow linking ‘Cancer Treatments’ to the ‘Post-Cancer Treatment Phase’ acknowledges that some cancer survivors, such as a significant proportion of those with advanced or metastatic cancer, will receive ongoing treatments and therefore not progress to the post treatment phase

Although a potential functional association between CRCI and psychosocial distress, as well as mood disorder symptoms, has been recognized, particularly in the *development* of CRCI, there has been limited acknowledgement of the broader potential etiological *contribution* that CRCI may make to the development and maintenance of the extensive range of psychopathology commonly observed in cancer survivors. Predominantly, existing literature on CRCI and psychopathology centers on two key areas: (1) the potential functional role that psychological distress, depression, and anxiety may play in the development of CRCI, and (2) the consequences of CRCI on quality of life and psychosocial distress. These explanatory mechanisms of action are fundamental to the development and provision of optimal prevention, treatment and supportive care approaches. However, we call for the increased acknowledgement and a concerted research effort into the potential *bidirectional and transdiagnostic* functional processes and association between CRCI and psychopathology in cancer survivors. In Fig. 1, we provide a high-level graphical depiction of our proposed functional associations between cancer (including the encompassing of the related established psychosocial stressors), cancer treatments, and psychopathology over the cancer continuum. Our proposition is not that CRCI singularly, or primarily, determines the development and perpetuation of psychopathology in cancer survivors. Instead, we suggest that CRCI may be one among several key factors that interact, contributing to the development and persistence of mental ill-health. A greater understanding of the potential functional association between CRCI and psychopathology and its intersectionality with other common systems has implications for risk identification, prevention, treatment, and supportive care approaches. For example, knowing the main pathways at play in cognitive processes (e.g. selective attention, memory, and repetitive negative thinking), and how they intersect with negative valence systems (e.g. exposure to threatening stimuli), social processes (e.g. affiliation and attachment), and arousal and regulatory systems (e.g. circadian rhythm) could lead to targeted early personalized approaches aimed at circumventing or attenuating risk for psychopathology.

The model is purposefully developed without an explicit time course to reflect that CRCI may develop at any point across the cancer trajectory [29]. This model therefore facilitates the proposition that CRCI may functionally contribute toward the development of psychopathology, and psychopathology may functionally contribute toward the development of CRCI, at any point in the cancer trajectory. Reflecting this proposition, assessment of cognition and mental health in cancer survivors should be ongoing, from initial diagnosis onward.

## Conclusion and research directions

Drawing upon non-oncology theoretical and empirical evidence concerning the functional association between cognition and psychopathology, we posit that individuals experiencing CRCI are likely at an elevated risk for the development and persistence of a broad spectrum of psychopathology. We call for future research to examine bidirectional functional relationships between psychopathology and CRCI, calling upon such frameworks such the RDoC. Further, in accordance with contemporary conceptualisations of psychopathology [21], specifically the HiTOP model [12], these examinations should include hierarchical dimensional approaches to assessing psychopathology. This includes the consideration of higher and lower-level domains, moving beyond a sole reliance on a traditional diagnostic approach. To develop the body of literature in this area approaches used can include more practical cross-sectional research, but also importantly longitudinal research using existing national and international datasets, as well as methods such as ecological momentary assessment, and mobile assessments. Experimental data may also be used to further strengthen our understanding. An additional area of consideration is the objective versus subjective assessment of cognition within cancer survivors. As there is not a strong relationship between subjective and objective assessments of cognition [29, 30], it is possible that these proposed relationships between cognition and psychopathology might be established using one assessment approach, but not the other. While cross-sectional research suggests that both objective and subjective measures of cognition show significant relationships with mental health in cancer survivors [28, 29], future research should use both subjective and objective measures when testing this proposed model, as this may inform future risk identification methodologies. Should a functional bidirectional relationship be established, it is imperative to develop an understanding of the specific domains and levels within cognition and psychopathology that are implicated, as well as how the cognitive processes intersect with other transdiagnostic domains. For example, while within non-cancer populations speed of processing seems especially related to transdiagnostic psychopathology across levels of analysis [11], whether this phenomenon is reflected within cancer survivors is unknown. Furthermore, an understanding of the associated time course is crucial. Subsequently, this knowledge can be used to inform risk identification, prevention, treatment, and supportive care approaches. Finally, these theoretical associations may underscore the imperative of the assessment of CRCI-related needs and the provision of optimal supportive care, which may reduce the risk of psychopathology development and persistence in cancer survivors [27, 28].

## Data Availability

Not applicable.
